# Evaluation of aflibercept and ziv-aflibercept binding affinity to vascular endothelial growth factor, stability and sterility after compounding

**DOI:** 10.1186/s40942-018-0143-x

**Published:** 2018-10-24

**Authors:** Julia de Lima Farah, Ronaldo Sano, Ieda Maria Longo Maugéri, Daniela Teixeira, Mayari Eika Ishimura, Gabriela Martins, Lycia M. J. Mimica, Cely Barreto da Silva, Carsten H. Meyer, João Rafael de Oliveira Dias, Gabriel Costa de Andrade, Michel Eid Farah

**Affiliations:** 1 Department of Ophthalmology, Santa Casa de Misericórdia of São Paulo, Rua Dr. Cesario Mota Junior 112, São Paulo, SP 01221-020 Brazil; 2 Department of Microbiology, Immunology and Parasitology, Paulista School of Medicine, Federal University of São Paulo, São Paulo, SP Brazil; 3 Department of Microbiology, Santa Casa de Misericórdia of São Paulo, São Paulo, SP Brazil; 4 Vision Institute, Department of Ophthalmology, Federal University of São Paulo, São Paulo, SP Brazil

**Keywords:** Retina, Anti-VEGF, Aflibercept, Ziv-aflibercept, Compounding

## Abstract

**Purpose:**

To investigate the binding affinity, stability, and sterility of aflibercept and ziv-aflibercept to vascular endothelial growth factor (Holash et al. in Proc Natl Acad Sci USA 99(17):11393–11398, 2002. 10.1073/pnas.172398299) after compounding and storage for up to 28 days at 4 °C and − 8 °C.

**Methods:**

Tuberculin-type 1-mL syringes were prepared containing aflibercept (40 mg/mL) and ziv-aflibercept (25 mg/mL). Samples were stored at 4 °C and − 8 °C for 0, 14, and 28 days and evaluated for the binding affinity of anti-VEGF to VEGF and stability using enzyme-linked immunosorbent assays. The evaluation of sample sterility was performed.

**Results:**

Laboratory trials with aflibercept and ziv-aflibercept showed preservation of the drug-binding capability to recombinant VEGF when stored in plastic syringes for up to 28 days at 4 °C and − 8 °C. No significant decrease in mass or concentration were observed. Microbiologic evaluations did not detect contamination in the syringes.

**Conclusions:**

The current study corroborates that compounded anti-VEGF drugs aflibercept and ziv-aflibercept do not loose stability or binding affinity and do not become contaminated if prepared under sterile conditions and stored at 4 °C or − 8 °C for 14 or 28 days.

## Background

Anti-vascular endothelial growth factor (VEGF) therapy became available and revolutionized the treatment of retinal disorders related to angiogenesis [2]. These drugs reduce vascular permeability and growth and leakage of new vessels involved in choroidal and retinal neovascularization [14, 16].

Anti-VEGF drugs used in Ophthalmology include an aptamer (pegaptanib, Macugen, Bausch & Lomb, Rochester, NY), a humanized monoclonal antibody (bevacizumab, Avastin, Genentech, Inc., South San Francisco, CA), an antibody fragment (ranibizumab, Lucentis, Genentech Inc.), and fusion proteins (aflibercept, Eylea, Regeneron Pharmaceuticals, Tarrytown, NY, and Bayer, Leverkusen, Germany), conbercept (Kanghong Biotech, Chengdu, China), and ziv-aflibercept (Zaltrap, Sanofi-Aventis, Bridgewater, NJ, and Regeneron Pharmaceuticals).

Aflibercept is a recombinant fusion protein engineered by the fusion of two extracellular domains of VEGF receptors 1 and 2 of human VEGF to the Fc portion of human immunoglobulin G1. In contrast to other monoclonal antibodies, aflibercept binds to VEGF-A, VEGF-B, and placental growth factor (PlGF). Aflibercept binds to VEGF-A with a tenfold higher affinity than other commercially available monoclonal antibodies. The highest VEGF-A binding affinity and the VEGF-B and PlGF binding capacity make this drug potentially more beneficial compared to bevacizumab and ranibizumab [21, 23].

The molecule of ziv-aflibercept is identical to that of aflibercept but at a lower concentration (ziv-aflibercept, 25 mg/mL; aflibercept, 40 mg/mL) and different osmolarities (ziv-aflibercept, 1000 mOsm/kg; aflibercept, 300 mOsm/kg) [24]. Aflibercept is isosmolar compared to the human vitreous and undergoes a different purification process, while ziv-aflibercept is hyperosmolar compared to the human vitreous [8, 29]. In 2012, the US Food and Drug Administration approved intravenously administered ziv-aflibercept for treating colorectal carcinoma refractory to or progressive after an oxaliplatin regimen [8, 29] and is not approved for intravitreal administration. However, studies from many countries have suggested that the drug might be a safe and effective alternative intravitreal treatment for retinal disorders [1, 3–5, 9, 11–13, 24, 25, 34].

Anti-VEGF drugs are used worldwide with good clinical results and are commercially available in sterile vials. According to the manufacturers’ instructions, the drugs should be stored between 2° and 8°, and the medications should not be frozen. Each vial has a total volume higher than the recommended dose for intravitreal administration (0.05–0.08 mL). Besides, for aflibercept and ranibizumab, approved for intravitreal administration, a single-use vial is recommended, although some ophthalmologists use the same vial for from two to four patients. For bevacizumab and ziv-aflibercept, which are used off-label for intravitreal administration, one vial can be used for up to 40 patients, excluding the residual volume in the vials, needles, or syringes.

Due to the clinical applicability of these drugs and high cost, the use of lower cost compounded single-use vials is important. Compounding is the subdivision of the packaging of a drug into individual parts to facilitate the availability of the amount established by the medical prescription [32] to reduce the treatment cost.

Precautions must be taken during compounding to perform this procedure in a sterile manner, maintain the sterility after compounding and storage, and maintain the biologic activity of the medication.

Ocular complications after intravitreal injections are well described, including infectious endophthalmitis. The reported incidence rates of infectious endophthalmitis after intravitreal anti-VEGF range from 0.02 to 0.08%. Studies have shown that the compounding of anti-VEGF drugs, assuming that regulatory surveillance was followed strictly, is unrelated to increases in ocular infectious complications [20].

The maintenance of the biologic activity of the medication can be assessed using specific assays such as enzyme-linked immunosorbent assay (ELISA) to determine the VEGF binding affinity and recovery of aflibercept and ziv-aflibercept after storage in syringes. Therefore, it would be possible to analyze the stability and sterility after compounding and storage under different temperature conditions (4 °C and − 8 °C) for up to 28 days.

## Methods

This experimental study to evaluate the binding in vitro affinity of commercially available antibodies to VEGF, loss of structure, and sterility under different storage conditions was performed in the laboratory of the Discipline of Immunology of the Department of Microbiology, Immunology and Parasitology of the Federal University of São Paulo–Paulista Medical School (UNIFESP/EPM) and the laboratory of Microbiology of the Department of Microbiology in the Department of Pathology of the Medical Sciences Faculty of Santa Casa of São Paulo from January to May 2017.

### Samples

Under sterile conditions, 1-mL tuberculin-type syringes were prepared with 0.05 mL of aflibercept (40 mg/mL) or 0.08 mL of ziv-aflibercept (25 mg/mL). Samples were stored at 4 °C to − 8 °C for 14 or 28 days. For each drug, three syringes were prepared for each medication as replicates of each condition. A syringe also was prepared for each medication before the assays and was referred as pre-assay. The preparation of the syringes and the experimental assays described below were carried out at the Immunology Division of Microbiology, Immunology and Parasitology Department of Federal University of São Paulo (UNIFESP/EPM).

### Anti-VEGF recovery of aflibercept and ziv-aflibercept after different storage conditions

The ELISA designed to detect anti-VEGF antibodies, a human monoclonal IgG, of which aflibercept and ziv-aflibercept are constituted, was performed as described by Dib et al. [15]. This assay evaluated the recovery of anti-VEGF drugs after the drugs were stored in polypropylene syringes for 0, 14, or 28 days at 4 °C and − 8 °C. Briefly, each sample obtained from the syringes was added to the ELISA 96-well plates (High Binding, Corning, NY) in replicates (50 µL/well) of the samples diluted in 0.9% saline (1:256,000, for aflibercept; 1:128,000, for ziv-aflibercept). The standard curves of aflibercept and ziv-aflibercept were performed by serial dilution in saline, starting at 1:64,000 dilution. These standard curves and the initial dilution of the medications had been standardized previously. The plates were incubated overnight at 37 °C. After washing with phosphate-buffered saline (PBS) with Tween 0.05% (PBST, Tween 20, Synth Diadema, SP, Brazil), 150 µL/well of blocking buffer solution (PBS-bovine serum albumin 1% [BSA], Sigma, St. Louis, MO) was added to eliminate the non-specific sites, and the plates were incubated for 1 h at room temperature. After washing, 50 µL of horseradish peroxidase (HRP)-conjugated anti-human IgG was added to each well at a dilution of 1:10,000 (KPL, SeraCare Life Sciences, MA, EUA) for 1 h at room temperature. Plates were washed and the enzymatic reaction was developed by the addition of 1 mg/mL of o-phenylenediamine (OPD, Sigma) diluted in phosphate-citrate buffer containing 0.03% of hydrogen peroxide (100 µL/well). The reaction was interrupted with the addition of 50 µL of 4 N sulfuric acid (Dinamica Química Contemporânea LTDA, SP, Brazil) solution/well. The optical density (OD) was measured at 492 nm with an ELISA reader (EnSpire, Multimode Plate Reader, PerkinElmer do Brasil, SP, Brazil). Anti-VEGF antibody was detected in each sample by concentration (µg/mL), based on the respective standard curve, calculated using GraphPad Prism version 5.0 software (GraphPad Software Inc., La Jolla, CA), and converted to recovered mass (µg).

### Aflibercept and ziv-aflibercept VEGF binding affinity under different storage conditions

The binding affinity evaluation of commercially available anti-VEGF antibodies (aflibercept and ziv-aflibercept) was performed using a standardized ELISA. (Nunes [26] unpublished) ELISA plates (High Binding) were coated with 1 µg/mL (50 µL/well) of capture antibody specific to human VEGF (R&D Systems Inc., Minneapolis, MN), in PBS and incubated overnight at room temperature. After washing with PBST, 150 µL/well of PBS-BSA 1% was added to eliminate non-specific biding sites for 1 h at room temperature. After this period and washing, 50 µL/well of human recombinant VEGF (R&D Systems Inc.) was added at 15,000 pg/mL and followed by incubation for 2 h at room temperature. Meanwhile, during the last hour of incubation, the samples obtained from the syringes were maintained at 37 °C. After washing, 50 µL/well of the samples was diluted in saline and added in replicates at 1:200,000 dilution for aflibercept and 1:100,000 dilution for ziv-aflibercept. To determine a concentration curve, serial dilutions in saline of aflibercept and ziv-aflibercept in a ratio of 1:2 starting at 1:50,000 were performed. This concentration curve was standardized previously, thus defining the initial dilution of the drugs. This was followed by an additional incubation at 37 °C for 2 h. After washing the plates, 50 µL of HRP-conjugated anti-human IgG (KPL) was added at the dilution of 1:5,000. After incubation at room temperature for 1 h and washing, the enzymatic reaction was performed as described previously to determine the OD at 492 nm. The binding affinity of anti-VEGF to the recombinant human VEGF molecule was detected in each sample by concentration (µg/mL), based on the respective standard curve, calculated using GraphPad Prism version 5.0 software.

### Sample sterility

For each syringe of aflibercept and ziv-aflibercept and storage conditions of 4 °C and − 8 °C, 12 µL of the stored material was inoculated on sterile filter paper discs 6 mm in diameter. The discs, which were completely saturated with the drugs, were placed on blood agar plates for 5 days and stored at 35 ± 2 °C. This procedure was performed under aseptic conditions and an unsaturated disc was incubated under the same conditions as a control.

### Data analysis

The statistical differences between samples were analyzed by one-way analysis of variance followed by Bonferroni’s multiple comparisons test using GraphPad Prism version 5.0 software.

## Results

Polypropylene syringes were prepared with aflibercept and ziv-aflibercept. To evaluate the amount of recovered anti-VEGF antibody after storage for different times and at different temperatures, an ELISA assay was performed to determine the mass of the anti-VEGF antibody present in the stored syringes and in the pre-assay syringe.

Table 1 shows that the pre-assay syringe of aflibercept showed no significant loss of anti-VEGF antibody (2.03 mg) compared to the expected mass (2.05 mg), according to the initial concentration and subsequent dilutions. There was also no difference in the anti-VEGF recovery when aflibercept was stored after compounding (Table 1). After storage for 14 days at 4 °C and − 8 °C, the measured aflibercept masses were 2.08 and 2.21 mg, respectively; after 28 days, 2.09 and 1.97 mg were detected, respectively, for 4 °C and − 8 °C.

**Table 1:** Anti-VEGF antibody recovered mass in aflibercept and ziv-aflibercept subjected to different storage conditions

	Anti-VEGF recovered mass (mg)	
	Aflibercept	Ziv-aflibercept
Expected mass	2.05	1.28
Pre-assay	2.03 ± 0.02	1.33 ± 0.01
14 days/4 °C	2.08 ± 0.10	1.17 ± 0.14
14 days/− 8 °C	2.21 ± 0.02	1.26 ± 0.11
28 days/4 °C	2.09 ± 0.17	1.28 ± 0.00
28 days/− 8 °C	1.97 ± 0.10	1.25 ± 0.06

Different samples of aflibercept and ziv-aflibercept were added to ELISA plates and detected by HRP-conjugated anti-human IgG and H_2_O_2_-OPD substrate. Anti-VEGF quantification (mg) was based on a standard curve performed with both drugs. The drugs were initially diluted in saline (1:256,000 for aflibercept; 1:128,000 for ziv-aflibercept). Statistical analysis was performed comparing the samples to the moment condition of each drug. Representative result of three experiments (mean)

The data are expressed as the mean ± SD

Similarly, there was no significant loss of mass for ziv-aflibercept in the pre-assay syringe prepared (1.33 mg) compared to the anti-VEGF mass expected to be measured (1.28 mg) (Table 1). Regardless of the storage conditions, there was no significant difference in the measured anti-VEGF antibody (Table 1) compared to the pre-assay condition stored at 4 °C or − 8 °C, for 14 (1.17 and 1.26 mg, respectively) or 28 days (1.28 and 1.25 mg, respectively).

Table 1 shows that the masses of the anti-VEGF antibody recovered from each syringe did not differ significantly compared to the masses of the pre-assay syringe independent of the storage conditions of aflibercept (*p* = 0.32) and ziv-aflibercept (*p* = 0.50).

To evaluate if the different storage times and temperatures altered the binding affinity of these drugs to the VEGF molecule, an ELISA was performed to determine the concentration of anti-VEGF antibody able to bind to the recombinant human VEGF molecule.

For each drug, an expected concentration was estimated according to the initial concentration and subsequent dilutions, which was calculated as 0.200 μg/mL for aflibercept and 0.125 μg/mL for ziv-aflibercept (Table 2). For aflibercept, the concentration of anti-VEGF antibody detected in the pre-assay syringe (0.208 μg/mL) did not differ significantly from its expected concentration or when stored for 14 days at 4 °C (0.185 μg/mL), at − 8 °C (0.196 μg/mL), 28 days at 4 °C (0.175 μg/mL), or at − 8 °C (0.147 μg/mL) (Table 2). Similarly, for ziv-aflibercept, the pre-assay condition (0.136 μg/mL) also did not differ significantly either from the expected concentration or from the different storage conditions for 14 or 28 days, at 4 °C (0.121 or 0.129 μg/mL, respectively) or − 8 °C (0.130 or 0.134 μg/mL) (Table 2).

**Table 2:** Evaluation of VEGF binding affinity in aflibercept and ziv-aflibercept samples under different storage conditions

	VEGF binding affinity (µg/mL)	
	Aflibercept	Ziv-aflibercept
Expected mass	0.200	0.125
Pre-assay	0.208 ± 0.041	0.136 ± 0.004
14 days/4 °C	0.185 ± 0.036	0.121 ± 0.005
14 days/− 8 °C	0.196 ± 0.010	0.130 ± 0.006
28 days/4 °C	0.175 ± 0.019	0.129 ± 0.013
28 days/− 8 °C	0.147 ± 0.014	0.134 ± 0.003

Samples of aflibercept and ziv-aflibercept were added to ELISA plates, after coating the plates with anti-VEGF capture antibody and after binding the recombinant molecule of human VEGF (both R&D components). The aflibercept’s or ziv-aflibercept’s anti-VEGF able to bind to the recombinant VEGF was detected by HRP-conjugated anti-human IgG and H_2_O_2_-OPD substrate, and quantification (µg/mL) was based on a standard curve performed with both drugs. The drugs were initially diluted in saline (1:200,000 for aflibercept; 1:100,000 for ziv-aflibercept). Statistical analysis was performed comparing the samples to the moment condition of each drug

The data are expressed as the mean ± SD

Table 2 shows that for both drugs regardless of the storage conditions, the concentrations of the anti-VEGF antibody detected did not differ significantly compared to that in the pre-assay syringe, indicating that there was no difference in the anti-VEGF affinity to the VEGF molecule with aflibercept (*p* = 0.14) or ziv-aflibercept (*p* = 0.15).

The cultures of the syringe samples seeded in a blood agar plate under all conditions and storage time were negative.

## Discussion

Anti-VEGF drugs are an important therapeutic arsenal that can be used to treat several eye diseases, mainly retinal disorders, most of which are chronic and degenerative. The therapeutic regimen requires several intravitreal applications of anti-VEGF drugs. The cost of the treatment is high for the patient and health system. Considering that these medicines can be injected nine to 11 times during year 1 of treatment (average, 17 times over 5 years), the related total costs and risks can be substantial [14, 16].

Compounding seems to be a possible way to reduce the cost, because the precise dose is administered, which reduces waste. However, administration of the compounded medication should maintain the safety and effectiveness of the treatment. In the current study, the in vitro effectiveness was assessed by analyzing the amount of recovered mass and binding capacity of the anti-VEGF drugs over time after compounding of the medications. The safety of compounding was assessed by checking the sterility of the compounded medications.

Several studies have reported the safety, sterility, and stability of different anti-VEGF drugs after compounding and storage [7, 10, 20, 30, 33, 35]. Chen et al. [7] suggested that the content of the bevacizumab vials in multiple doses at 4 °C remains sterile and the anti-VEGF activity is stable for up to 6 months. Nunes et al. confirmed the stability and sterility of bevacizumab for up to 30 days after compounding in polypropylene syringe at − 8 °C and 4 °C (Nunes [26], unpublished). Cao et al. [27] reported that compounding of ranibizumab and aflibercept in plastic syringes and storage for up to 4 weeks did not appear detrimental to the in vitro functional activity of these drugs. Mansour et al. [25] showed that ziv-aflibercept did not lose its anti-VEGF activity when stored at 4 °C in polycarbonate syringes for 4 weeks. Given this scenario, the compounding of anti-VEGF has been adopted in several centers around the world.

The immunoenzymatic assay was carried out on the samples from each syringe to evaluate if the masses of aflibercept and ziv-aflibercept decreased. No significant loss of anti-VEGF mass was found when we compared the pre-assay syringe to the syringes stored for 14 and 28 days at the two different temperatures.

The second ELISA evaluated the binding affinity of both drugs to recombinant VEGF 14 and 28 days after compounding and after storage at different temperatures. No changes in the VEGF binding affinity was seen in these samples compared to the binding affinity of the pre-assay syringe, indicating that the in vitro ability of the anti-VEGF molecules to bind to their respective receptors was preserved.

The current study corroborates what is described in the literature, i.e., that compounded aflibercept and ziv-aflibercept do not lose their stability and binding affinity and are not contaminated if prepared under sterile conditions and stored at 4 °C or − 8 °C for 14 or 28 days.

A difference from most studies in which compounded aflibercept was evaluated was that in the current study the tests also were performed at − 8 °C. Previous studies of drug storage and the manufacturer’s instructions have suggested that the drugs be stored between 2 and 8 °C (refrigerator) and not frozen [7, 22, 27]. However, in the current study we found that for up to 28 days aflibercept and ziv-aflibercept stored at − 8 °C did not lose mass or have decreased binding affinity compared to the pre-assay condition, when both medications were maintained according to the manufacturer’s instructions (at 4 °C). These data suggested that compounded aflibercept and ziv-aflibercept can be stored for up to 28 days in polypropylene syringes at 4 °C and to − 8 °C.

The current protocols for treating age-related diseases determine the use of intravitreal injections monthly or almost monthly [6, 28]. Although the incidence of injection-related endophthalmitis is relatively low, the possible consequences of the infection can be disastrous. Previous published reports have described cases of outbreaks of infections secondary to drug manipulation, not just for ophthalmologic use [20]. In 2011, the FDA issued an alert regarding cases of contamination that developed after use of intraocular bevacizumab at ophthalmology clinics in the US [17]. However, all patients were treated from the same drug batch compounded in the same pharmacy. *Streptococcus mitis/oralis* were the infectious agents present in most infected patients and all compounded syringes of the same batch that were unused [18].

The contamination of the syringes of the compounded drug in specific pharmacies was caused by breaks in the basic protocols of medication manipulation [19, 31], which emphasizes the importance of adopting strict protocols for the compounding process to avoid outbreaks or infections.

The current study showed that no syringes had microbial contamination in the microbiologic evaluations performed during all storage periods at both temperatures. The importance of assessing the stability and safety of these compounded drugs is essential to a possible cost-effectiveness analysis when comparing the medications studied here with other commercially available medications. Demonstration of the in vitro stability of aflibercept and ziv-aflibercept after compounding and storage for up to 28 days should be followed by further studies to address the possible effects of in vivo functional activity.
